# Swarms of Enzymatic Nanobots for Efficient Gene Delivery

**DOI:** 10.1021/acsami.4c08770

**Published:** 2024-08-27

**Authors:** Juan C. Fraire, Carles Prado-Morales, Ana Aldaz Sagredo, Ainhoa G. Caelles, Florencia Lezcano, Xander Peetroons, Anna C. Bakenecker, Valerio Di Carlo, Samuel Sánchez

**Affiliations:** † Institute for Bioengineering of Catalonia (IBEC), Barcelona Institute of Science and Technology (BIST), Baldiri i Reixac 10-12, 08028 Barcelona, Spain; ‡ Catalan Institute for Research and Advanced Studies (ICREA), Passeig de Lluís Companys 23, 08010 Barcelona, Spain; ⊥ Facultat de Farmàcia i Ciències de l’Alimentació, Universitat de Barcelona, 08028 Barcelona, Spain

**Keywords:** nanobots, enzyme catalysis, swarming, gene delivery, drug delivery, pDNA, transfection

## Abstract

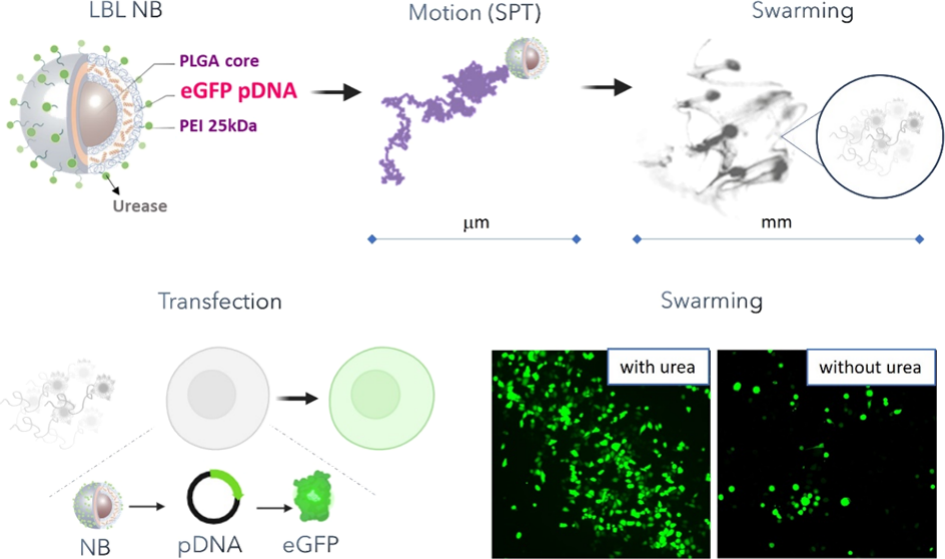

This study investigates the synthesis and optimization of nanobots (NBs) loaded with pDNA using the layer-by-layer (LBL) method and explores the impact of their collective motion on the transfection efficiency. NBs consist of biocompatible and biodegradable poly(lactic-*co*-glycolic acid) (PLGA) nanoparticles and are powered by the urease enzyme, enabling autonomous movement and collective swarming behavior. *In vitro* experiments were conducted to validate the delivery efficiency of fluorescently labeled NBs, using two-dimensional (2D) and three-dimensional (3D) cell models: murine urothelial carcinoma cell line (MB49) and spheroids from human urothelial bladder cancer cells (RT4). Swarms of pDNA-loaded NBs showed enhancements of 2.2- to 2.6-fold in delivery efficiency and 6.8- to 8.1-fold in material delivered compared to inhibited particles (inhibited enzyme) and the absence of fuel in a 2D cell culture. Additionally, efficient intracellular delivery of pDNA was demonstrated in both cell models by quantifying and visualizing the expression of eGFP. Swarms of NBs exhibited a >5-fold enhancement in transfection efficiency compared to the absence of fuel in a 2D culture, even surpassing the Lipofectamine 3000 commercial transfection agent (cationic lipid-mediated transfection). Swarms also demonstrated up to a 3.2-fold enhancement in the amount of material delivered in 3D spheroids compared to the absence of fuel. The successful transfection of 2D and 3D cell cultures using swarms of LBL PLGA NBs holds great potential for nucleic acid delivery in the context of bladder treatments.

## Introduction

Targeting the genetic bases of many diseases is rapidly being implemented, as demonstrated by the recent approval of various nucleic-acid-based therapeutics by the United States Food and Drug Administration (FDA) and the European Medicines Agency (EMA).^1^ The negatively charged and hydrophilic structure and the high molecular weight of nucleic acid therapeutics confer them with very poor cellular membrane permeability, low cellular uptake, as well as limited stability during blood circulation.^2^ The promise of nanotechnology-based drug delivery systems (DDSs) is to deliver nucleic acids selectively to the target tissues and cells with increased efficacy while reducing side effects. However, there are still remaining challenges for those DDSs, such as their improvement in terms of selective targeting, penetration of biological barriers, drug loading capacity, and efficient delivery on the subcellular level.^3^ These challenges are linked to the different physiological barriers that need to be overcome including extracellular barriers that are encountered prior to reaching the target cells (such as blood circulation, opsonization, endocytosis by the mononuclear phagocytic system, and tissue pressure),^4^ and also intracellular barriers such as crossing the plasma membrane and endosomal barriers.^5^ In that sense, nanoparticles (NPs) with an autonomous motion (nanomotors or nanobots—NBs) that have already demonstrated an enhanced active crossing of biological barriers^6,7^ and the delivery of therapeutic drugs into cells and tissues, have been proposed as the next generation of intelligent DDS platforms in nanomedicine.^8^ Enzyme-powered NBs are at the forefront, since they can utilize physiologically relevant fuels as their substrate and carry out catalytic reactions to power motion under *in vivo* conditions.^9^ These artificial nanomachines present self-propulsion at the nanoscale in the presence of the fuel, allowing them to overcome the medium viscous forces and Brownian motion.^10^ The possibility to use different enzymes as powering engines and also different carrier sizes and geometries has positioned these nanobots as a versatile and biocompatible alternative to generate self-propelling DDSs. Different enzymes have been explored, but recent studies have demonstrated that urease is arguably one of the most promising ones as it presents high catalytic rates that confer urease-functionalized nanobots with higher self-propelling capabilities compared to other enzymes.^11^ Moreover, their collective behavior (what is commonly referred to as swarming behavior) has been characterized *in vitro* demonstrating enhanced fluid mixing, collective migration, and enhanced mobility of NBs in comparison to the control without fuel.^12,6^ In addition, *in vivo* results demonstrated homogeneous distribution of nanobots after intravesical instillation in the bladder of mice in the presence of fuel, indicating that self-propulsion promotes convection and mixing in living reservoirs.^12,13^ Recent results depict a clear preferential accumulation of NBs in the tumor tissue after intravesical injection in a bladder cancer mouse model.^14^

Despite the outstanding performance demonstrated by these systems, drug delivery studies with NBs have mainly focused so far on the delivery of small molecules. This is also true for urease-based NBs, where most of the studies demonstrating enhanced drug delivery are based on doxorubicin *in vitro* (a chemotherapy medication used to treat cancer),^15,16^ which is currently limited to inducing cell killing in cancer cell lines. In contrast to conventional small molecular drugs, which generally target proteins (like doxorubicin, which stabilizes the topoisomerase II complex),^17^ nucleic acid therapeutics can manipulate gene expression to produce therapeutic proteins or to reduce harmful ones. These make these drugs suitable not only for cancer but also for other pathologies with well-established genetic targets, including infectious diseases, immune diseases, and Mendelian disorders (including neurological disorders). The most explored therapeutic molecules for the modulation of gene expression include plasmid DNA (pDNA) and mRNA (mRNA) for induction of expression, and smaller short interfering RNA (siRNA) for post-transcriptional gene silencing.^18^

Here, we describe the synthesis and characterization of pDNA-loaded NBs, including pDNA loading capacity, urease attachment, and motion parameters (at the single-particle level as well as their collective behavior or swarming). Cellular uptake, transfection efficiency, and cell viability are evaluated in two-dimensional (2D) cell cultures and three-dimensional (3D) spheroids as a function of incubation time, nanobot concentration, and fuel concentration. Results confirmed the effective enhanced delivery in the presence of urea acting as a fuel. Additionally, this study demonstrates that longer incubation times and higher NB concentrations lead to considerable delivery enhancements in both types of cell cultures. Lastly, we demonstrate enhanced transfection by means of swarms of pDNA-loaded NBs. Together, these results show that NBs represent a promising platform for nucleic acid delivery. Taking advantage of the endogenous nature of the fuel needed to induce thrust and the natural abundance of urea in the urinary tract, these NBs hold significant therapeutic potential as carriers for future gene therapies targeting bladder-related conditions.

## Results and Discussion

### Design and Characterization of pDNA-Loaded PLGA NBs

**Figure 1:**
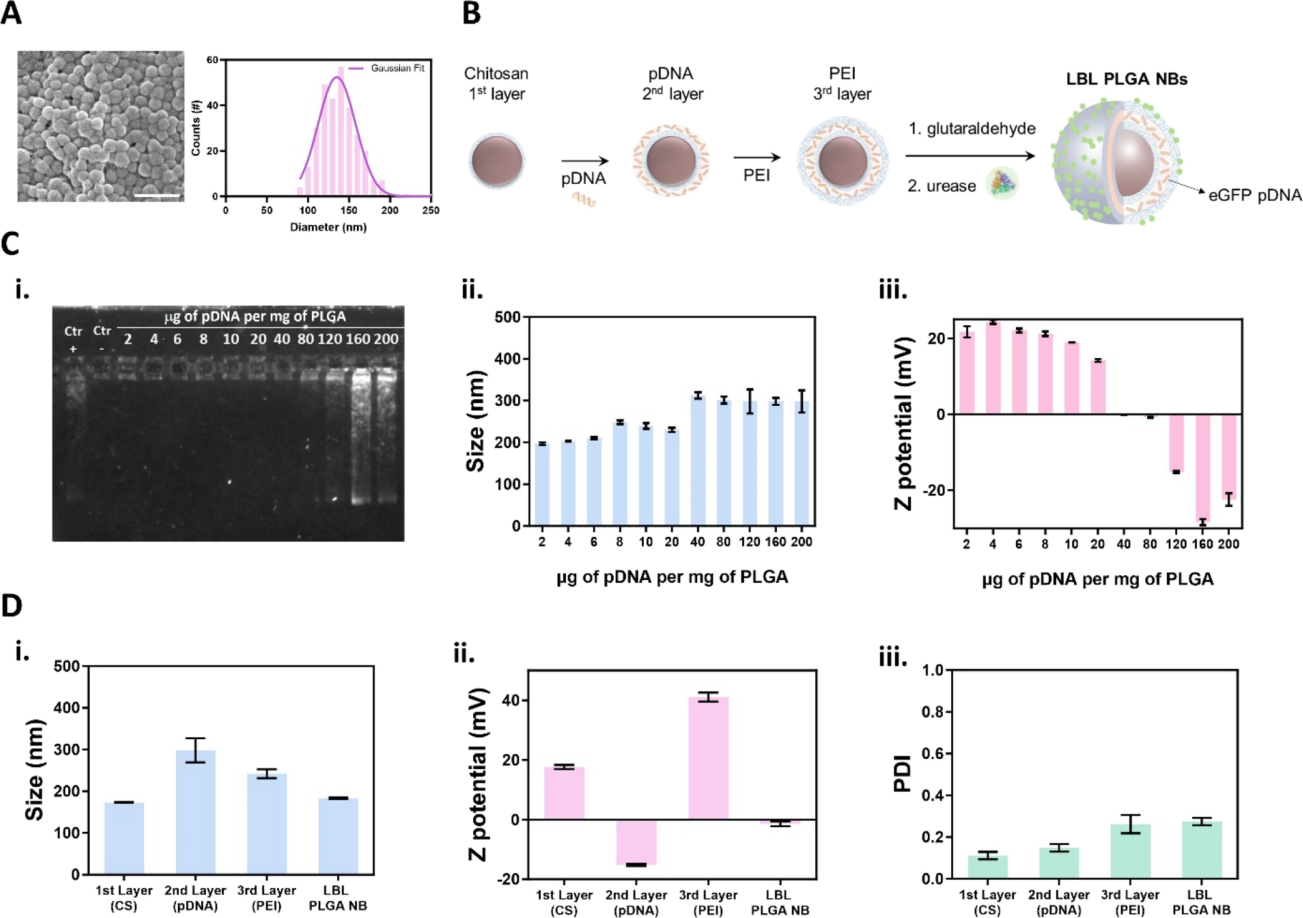
Design and synthesis of layer-by-layer pDNA-loaded urease-powered PLGA nanobots (LBL PLGA NBs). (A) Representative SEM image of PLGA NPs and size distribution of PLGA NPs determined by SEM analysis of 300 NPs from 3 different synthesis. The scale bar represents 400 nm. (B) PLGA NPs coated with chitosan (1st layer) were used for electrostatic loading of the negatively charged pDNA (2nd layer), which was further functionalized with PEI (3rd layer). LBL PLGA NPs were further functionalized with glutaraldehyde to form LBL PLGA@Glu, after which they were incubated in the presence of urease to finally form LBL PLGA NBs. (C) (i) Agarose gel of the supernatants collected from centrifugation and the physicochemical characterization of the pDNA loaded PLGA NPs by (i) dynamic light scattering and (ii) ζ-potential analysis after loading different pDNA/PLGA NPs mass ratios. (D) Physicochemical characterization of final LBL PLGA NBs and the different synthetic steps by (i) dynamic light scattering, (ii) ζ-potential analysis, and (iii) PDI.

In this study, we designed NBs based on nanocarriers consisting of poly(lactic-*co*-glycolic acid) (PLGA) NPs as a core. This polymer is already approved by regulatory agencies for use in humans due to its biodegradability and biocompatibility.^19^ These features make PLGA cores good candidates for the chassis of future nanobot-based formulations for gene therapy. PLGA NPs of 200 nm were synthesized by an oil-in-water emulsion in the presence of chitosan (CS), a linear biopolymer consisting of randomly repeating d-glucosamine and *N*-acetyl-d-glucosamine units. CS offers the advantage of being biodegradable and biocompatible while also being highly positively charged at a pH below the p*K*_a_. This allows it to easily form electrostatic complexes with nucleic acids.^20^ The scanning electron microscope (SEM) characterization and size distribution of the synthesized CS-PLGA NPs are shown in Figure 1A.

These NPs were used as scaffolds for an electrostatic assembly method based on the alternating adsorption of multivalent systems with complementary interactions. This method, known as layer-by-layer (LBL) assembly, creates highly controlled cargo delivery systems.^21^ This approach offers a versatile technique in terms of polymer and core composition and can be used to generate stable and efficient DDSs, as we previously demonstrated using gold and selenium nanoparticles.^22−25^ A schematic representation of the overall LBL design is shown in Figure 1B: CS-coated PLGA NPs (1st layer) were used to attach negatively charged pDNA (2nd layer). Afterward, a final polyethylenimine (PEI) layer (3rd layer) was applied to protect the pDNA and endow the particles with the presence of amino groups in the outer layer. This will allow the coupling of urease using glutaraldehyde cross-linking to obtain the final LBL PLGA NBs.

The nucleic acid loading capacity of the CS-PLGA NPs was evaluated by mixing pDNA encoding the expression of the green fluorescent protein (eGFP). For this, we added different mass ratios of pDNA to PLGA, ranging from 2 to 200 μg of pDNA per mg of PLGA. After complexation for 2 h, the supernatants corresponding to the different concentrations of pDNA were collected and studied through gel electrophoresis. A positive band in the gel would indicate an excess amount of pDNA, suggesting that the NPs are unable to conjugate such high pDNA concentrations.^22^ As shown in Figure 1C-i, intense pDNA bands are detected only for supernatants corresponding to higher amounts of pDNA (160 and 200 μg of pDNA). Therefore, the maximum concentration that can be loaded into the PLGA NPs is 120 μg per mg of PLGA. Additionally, the attachment of pDNA to form a stable second layer in the LBL process was also monitored by dynamic light scattering (DLS), revealing a gradual increase in the hydrodynamic diameter of the NPs (Figure 1C-ii), from 197 ± 3 nm after adding 2 μg of pDNA to 298 ± 12 nm after adding 200 μg. Surface loading of pDNA was also confirmed by the measured changes in ζ-potential starting at 21 ± 1 mV and decreasing to −22 ± 2 mV (Figure 1C-iii). Figure 1D shows the DLS characterization of the final LBL PLGA NBs, including the intermediates and the final synthesized motor. As can be seen in Figure 1D-i, a slight increase in hydrodynamic size was observed upon NB formation, from 173 ± 1 to 183 ± 2 nm. ζ-potential measurements shown in Figure 1D-ii depict the drastic changes in the surface charge during the different functionalization steps, with an overall change of Δ = −23 mV from the first layer to the final LBL NB. These changes in the ζ-potential, especially after the last functionalization step, indicate that the amine groups of the third layer (PEI) were successfully activated through glutaraldehyde chemistry, allowing urease functionalization. Polydispersity index (PDI) was slightly affected during the different steps of functionalization, increasing from 0.11 to 0.27 (Figure 1D-iii). The LBL formation was also characterized by Fourier transform infrared (FTIR) (Figure S1 in the Supporting Information). As can be seen in the new Figure S1, the spectra of the initial core particles (chitosan coated PLGA NPs) show clear vibrational modes that can be assigned undoubtedly to functional groups of either PLGA (*e.g.*, C=O at 1746 cm^–1^) or chitosan (*e.g.*, N–H at 1647 cm^–1^).^26^ By direct subtraction of the spectrum of LBL NPs (after subsequently loading first of pDNA, and second of PEI) the corresponding signals assigned to vibrational modes of PEI (*e.g.*, N–H at 1639 cm^–1^), thus confirming the successful coating with the third layer.

### Single-Particle Motion, Toxicity, and *In Vitro* Swarming of pDNA-Loaded PLGA NBs

Once the pDNA-loaded PLGA NBs were synthesized, we performed corresponding motion analysis using optical microscopy. An asymmetric distribution of enzymes around the NB surface, which occurs stochastically during the enzyme binding process, would allow us to observe self-diffusiophoresis in the presence of urea as fuel.^27,28^ In order to evaluate this, the NB’s motion trajectories were tracked in the absence and presence of different urea concentrations in 1× PBS: 0, 50, 100, 200, and 300 mM (see Figure S2A in the Supporting Information), with the last one being the upper limit as it is the maximum urea concentration found *in vivo* in the bladder.^29^ The obtained trajectories were analyzed using an in-house developed Python code to compute the mean squared displacement (MSD) as a function of time for each condition (Figure S2B). As can be seen in the graph, the MSD always increased linearly with time, corresponding to an enhanced diffusion regime, which is expected for particles with a small rotational diffusion time.^10^ The diffusion coefficients obtained from linearly fitting the MSD are shown in Figure S2C. As depicted in the graph, NBs showed a significant increase in the diffusion coefficient dependent on urea concentration, reaching a maximum of 2.1 ± 0.3 μm^2^/s at 300 mM of urea (an increase of 102%).

**Figure 2:**
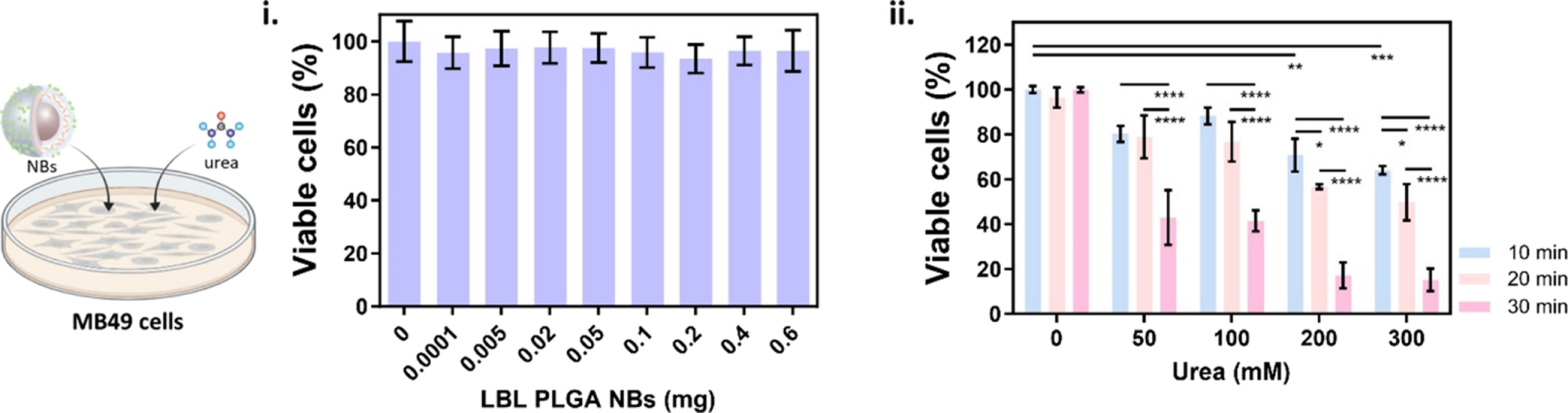
Cell toxicity of LBL PLGA NBs in MB49 cells (2D culture): (i) Viability as a function of LBL PLGA NBs concentration in PBS. Viability was assessed at 24 h after a 4 h incubation with PLGA NBs. No statistical significance was found between conditions (one-way ANOVA). (ii) Viability as a function of the incubation time for 0.02 mg of LBL PLGA NBs for increasing concentrations of urea in PBS. Cell viability was determined by CellTiter-Glo assay 2 h after treatment. Statistical significance (two-way ANOVA, with multiple comparisons) is indicated when appropriate (*p < 0.05, **p < 0.01, ***p < 0.001, ****p < 0.0001). Created with BioRender.com.

After characterization of the single-particle motion of the LBL PLGA NBs, clearly demonstrating their self-propelling capabilities, we then proceeded to evaluate their possible toxicity. Since these nanobots are powered by self-diffusiophoresis in the presence of urea as fuel, we chose to target the urinary tract for potential therapeutic applications. In this study, we utilized 2D cultures of MB49 cells (a commonly used murine bladder cancer cell line for *in vitro* studies) to evaluate the toxicity of the NBs. Our first step was to assess the potential toxicity of the NBs themselves by exposing MB49 cells to increasing concentrations of LBL PLGA NBs without fuel for a 4 h period. After the incubation, the cells were washed and further incubated in full cell culture medium for 24 h before assessing cell viability using the CellTiter-Glo metabolic assay. Figure 2i demonstrates that the toxicity observed across the concentration range of 0.0001 to 0.6 of NBs was negligible. Next, we investigated the effect of incubation time (10, 20, or 30 min) in the presence of increasing concentrations of urea in PBS (0, 50, 100, 200, or 300 mM) for a constant concentration of 0.02 mg of NBs. Figure 2ii shows that as the incubation time and urea concentration increase, there is a clear decrease in cell viability, which is known to be associated with the presence of byproducts from the enzymatic reaction, particularly ammonia.^16^ In order to minimize cell toxicity while taking advantage of the self-propelling capabilities of the NBs, we decided to proceed with experimental conditions that achieved viabilities ≥80%. Therefore, we set an upper limit of 20 min for incubation time and a 100 mM urea concentration.

**Figure 3:**
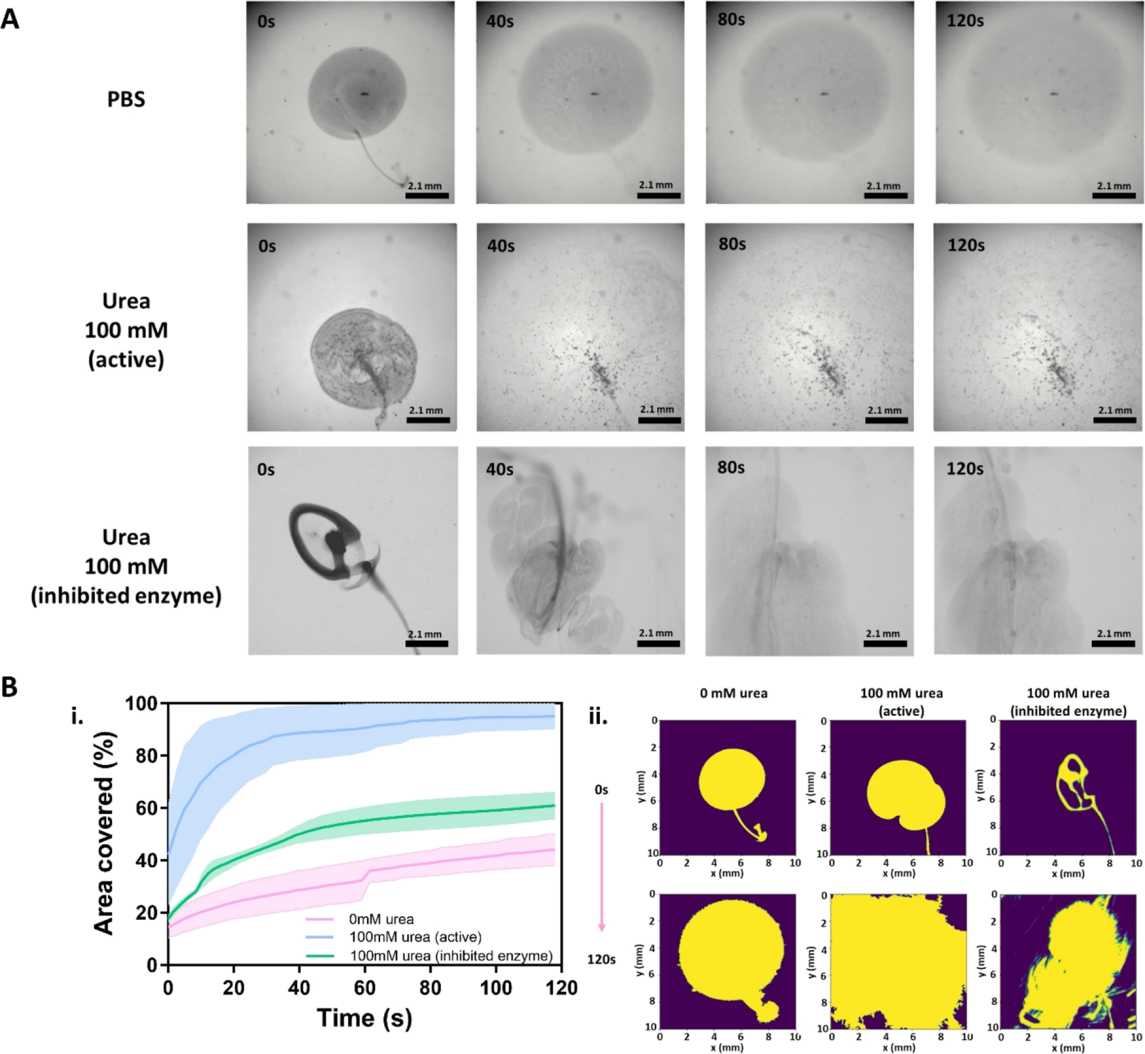
Optimization of *in vitro* swarming of LBL PLGA nanobots for delivery experiments. (A) *In vitro* swarming analysis of Swarm: snapshots of LBL PLGA NBs populations in PBS (top row) and in 100 mM urea in PBS (middle row). Snapshots of the population of inhibited particles in the presence of 100 mM urea are presented in the lower row. (B) Analysis of the *in vitro* swarming behavior of urease-powered LBL PLGA NBs. (i) Cumulative area covered by the swarm cloud as a function of time performed and (ii) snapshots of the effective area at the indicated time points (0 and 120 s). Swarming behavior was evaluated by adding a drop of 2 μL containing 0.02 mg of NBs for active bots in 0 and 100 mM urea in PBS, and for inhibited bots in 100 mM urea in PBS (*n* = 2).

Next, we evaluated the collective motion dynamics and swarming behavior of LBL PLGA NBs *in vitro* using optical microscopy. For these experiments, we placed 2 μL of NB dispersion (0.02 mg) in the center of a 3 mL Petri dish. The dish contained either PBS (control) or a 100 mM urea solution in PBS. We also included a control group with inhibited NBs in the presence of 100 mM urea. Inhibited NBs are the same LBL PLGA NBs, but they were incubated for 10 min in the presence of 1 mM acetohydroxamic acid, which inhibits the hydrolysis of urea reversibly. We recorded corresponding videos for 2 min (Figure 3A and Movie S1). In the absence of urea, the NBs showed relatively slow diffusion and sedimentation. However, in the presence of urea, we observed vigorous collective diffusion that further evolved into the formation of patterns with high particle densities. This collective behavior is attributed to buoyancy-induced convection resulting from the density difference between the NBs and the products of the enzymatic reaction with the fuel medium.^30^ Upon reaching the solid–air interface, it spreads along the interface, forming unstable fronts that further sink in the form of finger-like clusters or heaps of NBs (up-concentration of NBs). This collective behavior will be referred to in this study as swarm formation or swarming). Overall, the swarming behavior of NBs led to a rapid expansion that covered the entire field of view in less than 20 s. In the case of inhibited NBs, we observed the formation of “plums” at the seeding point where the drop was added. Over time, these plums collided and the NBs were deposited at the bottom of the dish.

To gain further insight into the dynamics of the swarm, we conducted a computational analysis to track the evolution of the swarm’s effective area (refer to Movie S2 in the Supporting Information). Figure 3B-i demonstrates that in the absence of fuel, or even for the inhibited NBs, nanobots slightly increase the effective area occupied by the swarm cloud over time. However, when active NBs are exposed to 100 mM urea, a significant change occurs in the area covered by the swarm within the first 20 s, reaching over 90% coverage of the field of view. This is further supported by comparing snapshots of the effective area at different time points (0 and 120 s), where a clear increase in the covered area can be observed in the case of swarms of active LBL PLGA NBs in the presence of urea (Figure 3B-ii). Similar results regarding the swarming effect were observed when working at 50 mM urea in PBS (Figure S3 in the Supporting Information and Movies S3 and S4), but the lower fuel concentration translated a more modest coverage, reaching 48%.

### Cellular Uptake and Viability in MB49 Cells

**Figure 4:**
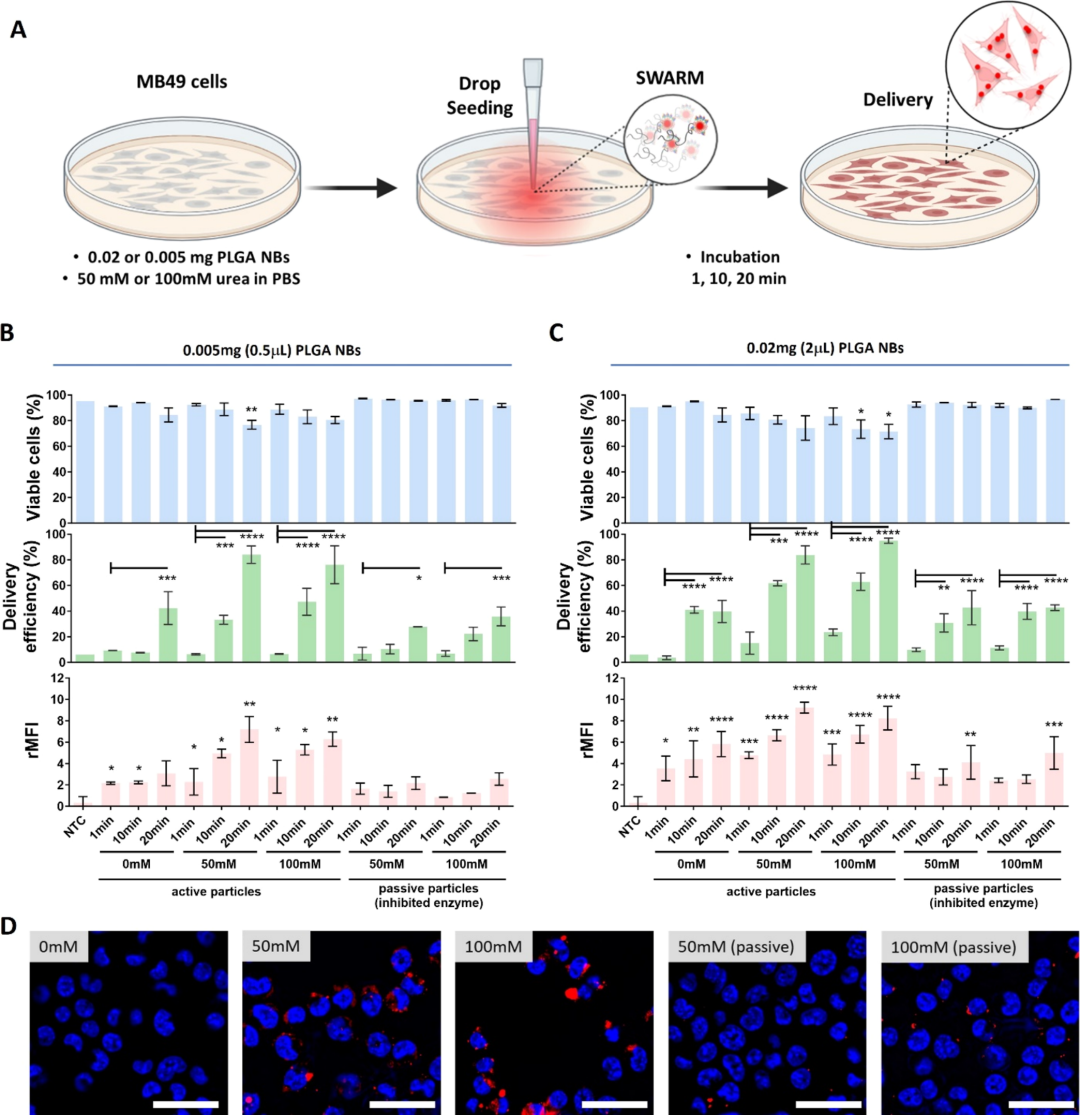
Delivery by swarms of Cy5-labeled LBL PLGA NBs in MB49 cells (2D culture). (A) Schematic overview of the experimental design for the evaluation of the delivery efficiency of swarms of LBL PLGA NBs. For swarming effect evaluation: a drop of 0.5 μL containing 0.005 mg or 2 μL containing 0.02 mg of Cy5-labeled LBL PLGA NBs was added in the center of a dish previously seeded with MB49 cells and filled with 50 mM or 100 mM urea in PBS. After adding the drop, the system was left to evolve undisturbed for 1, 10, or 20 min prior to quantifying the delivery efficiency. Delivery efficiency (% positive cells for the presence of NBs—green bars) and relative mean fluorescence intensity per cell (rMFI—pink bars) were determined by flow cytometry for (B) 0.5 μL containing 0.005 mg of NBs or (C) 2 μL containing 0.02 mg of NBs. Cell viability was determined by CellTiter-Glo assay in parallel (blue bars). All results are represented as mean ± SD for *n* = 3 biologically independent samples. Statistical significance (one-way ANOVA, with and without multiple comparisons) is indicated when appropriate (*p < 0.05, **p < 0.01, ***p < 0.001, ****p < 0.0001). (D) Representative microscopy images of MB49 cells 20 min after treatment with 0.02 mg of active or inhibited NBs at different urea concentrations in PBS. Nuclei were stained with Hoechst 33342 (blue), while Cy5-labeled NBs are shown in red. The scale bar corresponds to 100 μm. Created with BioRender.com.

We proceeded to evaluate the delivery efficiency of PLGA NBs in the MB49 cells. For this, we made use of Cy5-labeled PLGA NPs as the core, which allow the quantification of the delivery efficiency by flow cytometry. Cell viability was determined in parallel using the CellTiter-Glo metabolic assay. One important aspect of any delivery experiment with NBs is to be able to correlate the cell experiments with motion characterization. In that sense, we administered the NBs similarly to how it was done in the swarming experiments; *i.e.*, a drop of NBs was placed in the center of a 3 mL Petri dish. Figure 4A shows a schematic overview of these experiments: (i) the cell culture is filled with 3 mL of 0, 50, or 100 mM urea in PBS; (ii) a 0.5 μL (0.005 mg) or a 2 μL (0.02 mg) drop of NB dispersion is placed in the center of the Petri dish; (iii) the system is allowed to evolve undisturbed for a certain incubation time (1, 10, or 20 min), after which the cells are washed with PBS and supplemented with fresh cell medium for further analysis. Inhibited particles were evaluated in the presence of 50 and 100 nM urea in PBS as controls. Figure 4B,C shows the results obtained by adding 0.5 and 2 μL of NBs, respectively. As can be seen in the cell viability graph (blue bars), there is a slight decrease in cell viability as a function of the incubation time for active particles for both explored urea concentrations (50 and 100 mM urea), regardless of the effective concentration of NBs administered. No apparent toxicity was detected in the absence of fuel (0 mM urea). This was also the case for inhibited particles, irrespective of the fuel or NB concentration. Note that cell viability remained above >75% in all cases.

There are two parameters that can be determined regarding the delivery efficiency: (i) the percentage of delivery or delivery efficiency, which indicates the portion of the cell populations whose signal intensity lies above a certain threshold; and (ii) the relative mean fluorescence intensity, which indicates how much material has been delivered per cell compared to the control of nontreated cells (NTC). The delivery efficiency is presented in the middle graph (green bars) of Figure 4B,C for 0.5 and 2 μL of NBs, respectively. In every case, an increasing trend can be observed as a function of the incubation time, regardless of the fuel and NB concentration, and the active or passive nature of the NBs. Nevertheless, there is a clear enhancement in the delivery efficiency when using active NBs in the presence of urea due to the swarming collective behavior that can be rationalized considering the quick expansion and further deposition which leads, overall, to a better and faster distribution of particles in the timeframes of the experiment. Delivery efficiency, in the presence of 100 mM urea, reaches values of 84 and 95% for 0.005 and 0.02 mg NBs, respectively. This represents a delivery enhancement of 2.2- and 2.8-fold compared to the absence of fuel. Note that inhibited particles showed similar delivery efficiencies (35–40% delivery) compared to the control of active particles in the absence of fuel. This enhancement in the delivery efficiency associated with the formation of an explosive swarming effect was also observed for the amount of material delivered determined by the relative mean fluorescence intensity (rMFI). The calculated rMFI (pink bars) for 0.5 and 2 μL of NBs is presented in Figure 4B,C, respectively. The rMFI values consistently increase as the incubation time increases. It is worth noting that when active NBs are used in the presence of urea, there are significant improvements in the rMFI values compared to inhibited particles and the absence of fuel. These improvements result in 6.8- to 8.1-fold enhancements in the amount of material delivered. Based on the overall results of the delivery experiment on MB49 cells, it was decided to continue with the higher concentration of NBs (0.02 mg) and 20 min of incubation time to confirm the flow cytometry data with by light microscopy. Figure 4D shows representative microscopy images of the fluorescently labeled LBL PLGA NBs (red) in cells imaged 30 min (20 min incubation of NBs + 10 incubation with nuclei staining) after the addition of active nanobots in the absence of fuel as well as 50 and 100 mM urea in PBS. Inhibited NBs in the presence of the two concentrations of fuel explored were included as controls. As can be seen in the images, there is clear enhancement in the amount of internalized material in the presence of fuel (*i.e.*, in the conditions where swarming behavior is induced) compared to the absence of fuel or to the use of inhibited particles. This result clearly indicates that the swarming behavior of the active particles leads indeed to higher amounts of particles delivered per cell, confirming the results shown in Figure 4C.

When describing self-propelling particles as nanocarriers, it is important to quantitatively determine whether their motion provides a net advantage compared to that of inhibited particles. To easily compare different conditions (incubation time, NB and fuel concentration), we calculated the delivery enhancements as the ratio of active particles to inhibited particles, taking into account the percentage of the cell population (delivery efficiency) and the relative amount of material delivered (rMFI). The results in Figure S4 in the Supporting Information show that all conditions had ratio values >1, indicating a clear effect of the active particles. It is worth noting that this enhancement effect is more pronounced for the amount of material delivered per cell, especially at lower concentrations of NBs. This can be explained by the fact that at higher concentrations more particles saturate the endocytic machinery for the same number of cells. However, at lower concentrations, the swarming effect of active particles leads to a better distribution of particles, resulting in a higher percentage of the cell population testing positive for the presence of particles and a higher number of particles per cell. The collective behavior of urease-powered nanobots was described as buoyancy-induced convection as a consequence of introducing a drop of nanobots into a fuel medium.^30^ NBs exhibit directional upward movement due to buoyancy arising from the density difference between the byproducts of the enzymatic reaction and the fuel medium. When reaching the solid–air interface, the NBs spread along the interface, forming unstable fronts that further sinks in the form of finger-like clusters or heaps of NBs (up-concentration of NBs).^6,30^ We hypothesize that, as these heaps have a directionality toward the cell membrane with an attributed force, their collective displacement toward the cells will lead to a favorable accumulation of the particles onto the cells, resulting in the observed enhancement in the delivery efficiency. Overall, the enhancements observed for the LBL PLGA NBs reported here can be attributed to the formation of vigorous swarms that lead to a better distribution of particles due to the flows generated in the process, which further sink toward the cells in the form of heaps of NBs that mediate a better internalization in the cells.

Despite drug delivery accounts for more than 25% of reported applications of nano- and micromotors,^31^ most of the quantifications performed were only based on fluorescence intensity measurements or the analysis of microscopy images’ MFI. These methods are laborious and only provide semiquantitative data.^32^ A more quantitative approach to measuring the level of nanoparticle uptake in mammalian cells is through flow cytometry. In a previous study, Llopis-Lorente and co-workers evaluated the delivery efficiency of 400 nm urease-powered silica NBs in HeLa cells using flow cytometry.^15^ To the best of our knowledge, this is the only report on the use of enzyme-powered NBs that quantifies the uptake through this method. In their study, they reported enhancements of 1.2-fold in the delivery efficiency (percentage of the cell population positive for the presence of NBs) and 2.5-fold in the MFI (amount of material delivered per cell) when working with a 50 mM urea concentration and 1 h incubation time. It is important to note that in this study the swarming effect was not studied, and the reported effect is linked to the enhanced diffusion of individual NBs. When considering previous results obtained by exploiting enhanced diffusion in relation to the enhancements reported here due to swarming behavior (2.1-fold for delivery efficiency and 2.5-fold for rMFI), it can be concluded that there is a clear beneficial effect in terms of delivery enhancements.

### pDNA Transfection Efficiency in MB49 Cells

**Figure 5:**
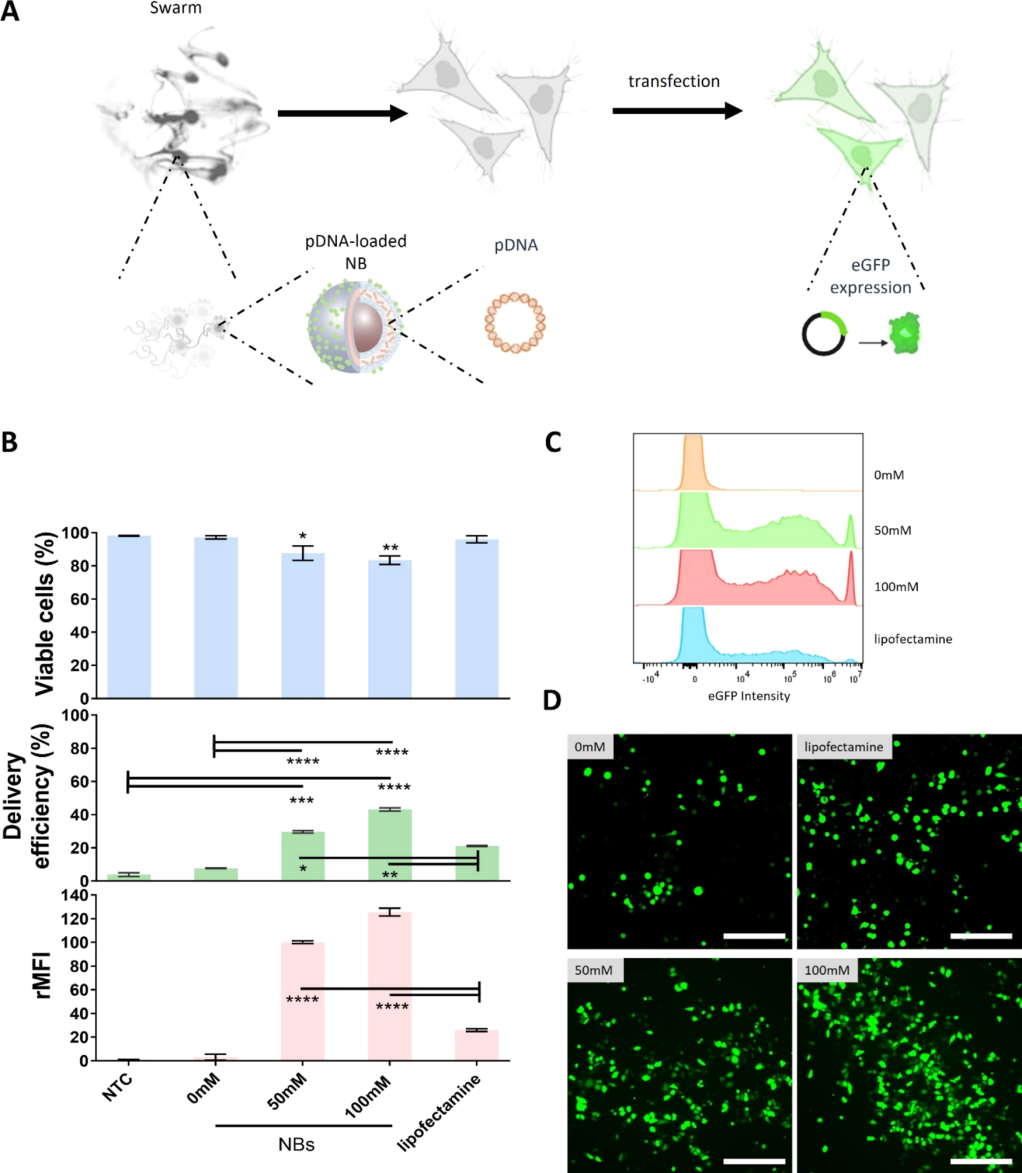
pDNA transfection by swarms of LBL PLGA NBs in MB49 cells (2D culture). (A) Schematic overview of the swarm-mediated transfection by delivery of pDNA encoding for eGFP. eGFP expression was evaluated 24 h after treatment. (B) Transfection efficiency (=% positive cells for eGFP expression—green bars) and relative mean fluorescence intensity per cell (rMFI—pink bars) were determined by flow cytometry for experiments performed by adding a drop of 2 μL containing 0.02 mg of NBs. Cell viability was determined by CellTiter-Glo assay in parallel (blue bars). Lipofection was included as a benchmark comparison using Lipofectamine 3000 loaded with 3.2 μg of pDNA (effective pDNA concentration in 0.02 mg of NBs). All results are represented as mean ± SD for *n* = 3 biologically independent samples. Statistical significance (one-way ANOVA, with and without multiple comparisons) is indicated when appropriate (*p < 0.05, **p < 0.01, ***p < 0.001, ****p < 0.0001). Representative (C) histograms of cell population distributions as a function of eGFP intensity obtained by flow cytometry, and representative (D) fluorescent microscopy images of transfected MB49 cells expressing eGFP (green signal) 24 h after treatment. The scale bar corresponds to 300 μm. Created with BioRender.com.

For the next step, we proceeded to evaluate the capability of LBL PLGA NBs in mediating the transfection of pDNA encoding eGFP expression (which represents a proof-of-concept model for the successful intracellular delivery of active macromolecules). For this, we used the optimized conditions obtained from delivery and viability measurements. Briefly, MB49 cells were exposed to 2 μL (0.02 mg) of NBs in the presence of 50 or 100 mM urea in PBS (Figure 5A). NBs preloaded with 120 μg of eGFP-pDNA per mg of PLGA were incubated for 20 min. After this period, the cells were washed with PBS, and a new full cell culture medium was added. The cells were further incubated for 24 h prior to the analysis of eGFP expression. We proceeded with only the control in the absence of fuel with active NBs, as the use of inhibited particles in the presence of fuel was demonstrated to not have a clear impact on the delivery efficiency, rMFI, or viability, giving similar values as the absence of fuel control (Figure 4C). The number of eGFP-positive cells was quantified using flow cytometry, while cell viability was determined in parallel using the CellTiter-Glo metabolic assay at 24 h post pDNA transfections. Figure 5B shows the overall pDNA transfection results, which include cell viability, transfection efficiency, and rMFI. Flow cytometry results indicated 30% eGFP-positive cells with a viability of 87% for experiments working at 50 mM urea in PBS. Moreover, the percentage of transfected cells could be further increased to 43% when using 100 mM urea without compromising cell viability (83%). In this case, the rMFI values also increased by more than 42-fold with respect to the control in the absence of fuel. We also performed a direct comparison in transfection efficiency between swarm-mediated delivery with NBs and the commonly used Lipofectamine 3000 transfection reagent.^33^ At the effective concentration of pDNA used in the NBs experiments (3.2 μg), lipofectamine gave 2-fold less transfection efficiency and 4.8-fold less rMFI than the use of active NBs in the presence of 100 mM urea. Figure 5C,D shows representative histograms of cell population distributions as a function of eGFP intensity obtained by flow cytometry and representative fluorescent microscopy images of transfected MB49 cells expressing eGFP (green signal) 24 h after treatment, respectively. The performance of the NBs as a function of fuel concentration and the comparison with respect to lipofectamine could be clearly evidenced in the number of cells expressing eGFP.

### Delivery and Transfection Efficiency in RT4 Cells

**Figure 6:**
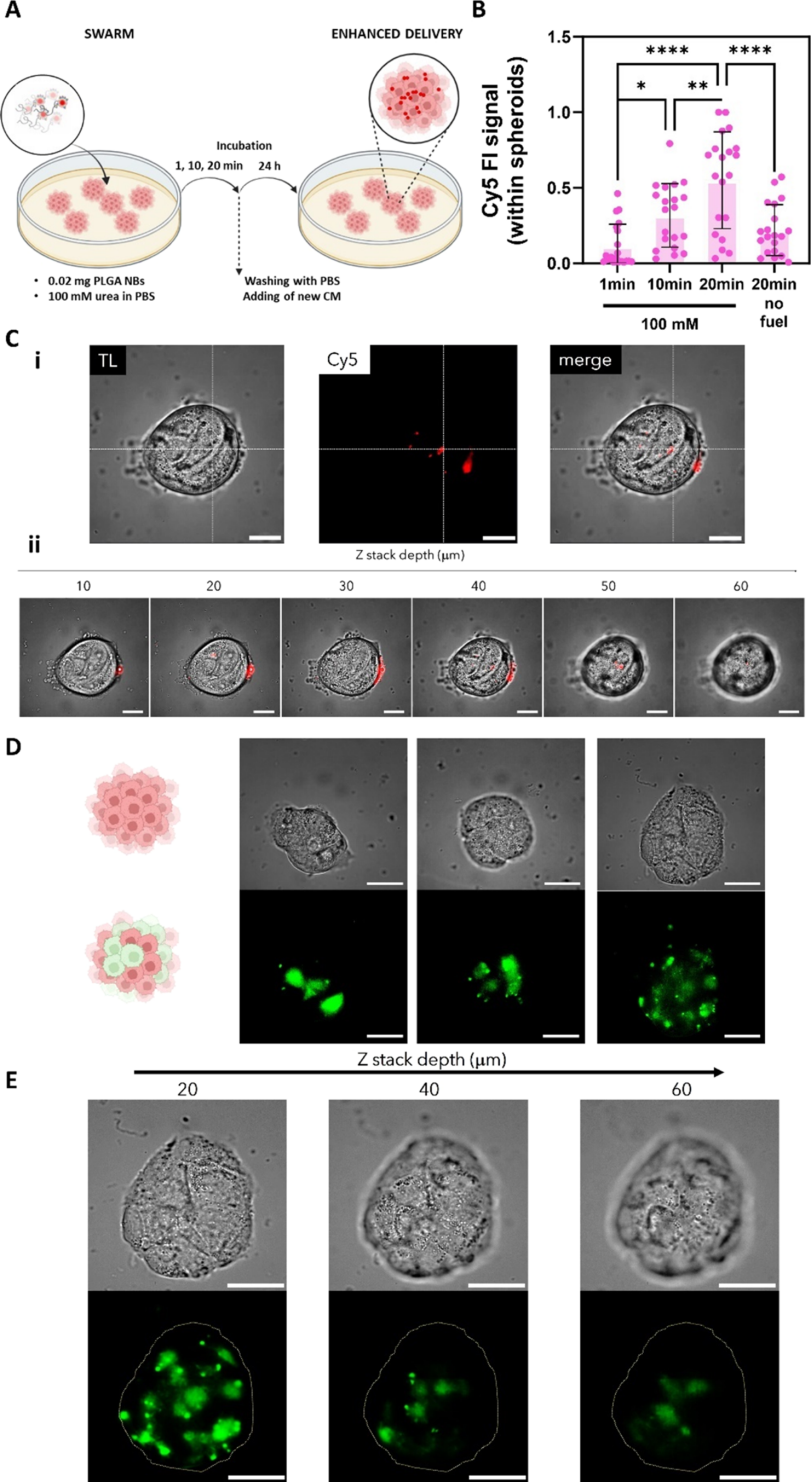
Delivery and transfection by swarms of LBL PLGA NBs in spheroids of human urinary bladder-derived RT4 cells (3D). (A) Schematic overview of the experimental design for the evaluation of the delivery efficiency of swarms of LBL PLGA NBs. For swarming effect evaluation: a drop of 2 μL containing 0.02 mg of Cy5-labeled LBL PLGA NBs was added in the center of a dish previously seeded with spheroids of RT4 cells and filled with 100 mM urea in PBS. After adding the drop the system was left to evolve undisturbed for 1, 10, or 20 min. After this period, the cells were washed with PBS and were incubated for 24 h in fresh medium prior to quantifying the delivery efficiency by fluorescence microscopy. (B) Normalized mean fluorescence intensity of the NB’s signal within RT4 spheroids at different incubation times. The condition of 20 min incubation in the absence of urea was included as a control. Results correspond to at least 20 spheroids per condition. Statistical significance (one-way ANOVA, with multiple comparisons) is indicated when appropriate (*p < 0.05, **p < 0.01, ***p < 0.001, ****p < 0.0001). (C) Microscopy images of: (i) the middle focal plane (transmission light, red channel showing Cy5-labeled NBs and merged image) and (ii) z-stack (merged image) of an RT4 spheroid after 20 min treatment with 0.02 mg of Cy5 labeled NBs in 100 mM urea. The scale bar corresponds in every case to 100 μm. (D) Representative fluorescent microscopy images and (E) z-stack of a transfected RT4 spheroid expressing eGFP (green signal) 24 h after treatment with 0.02 mg of pDNA-loaded LBL PLGA NBs. The scale bar corresponds to 100 μm. Created with BioRender.com.

In the previous section, we demonstrated that swarms of urease-powered NBs have the ability to enhance delivery and transfection in MB49 cells (2D cell culture). Despite the interesting insight that could be obtained from experiments performed using 2D cultures, there is great consensus on 3D cultures better mimicking real tumor environments in terms of cell morphology and physiology.^34^ For this reason, we proceeded to evaluate delivery and transfection using swarms of LBL PLGA NBs in 3D cultures (spheroids) of human urinary bladder transitional cell papilloma RT4 cells. This system presents an additional challenge in terms of overcoming intracellular barriers, as well as the extracellular matrix and cells’ tight junctions. Figure 6A shows a schematic overview of experiments: (i) the cell culture is filled with 3 mL of 0 or 100 mM urea in PBS; (ii) a 2 μL (0.02 mg) drop of NB dispersion is placed in the center of the Petri dish; (iii) the system is allowed to evolve undisturbed for a certain incubation time (1, 10, or 20 min), after which the spheroids are washed with PBS and supplemented with fresh cell medium and further incubated for 24h prior analysis. We quantified the fluorescence intensity in the red channel, which corresponds to the presence of Cy5-labeled LBL PLGA NBs. We chose this semiquantitative method to enable a direct comparison with previous reports using NBs, as the penetration of NBs within spheroids has only been analyzed through fluorescence intensity measurements (FI). Figure 6B displays the normalized Cy5 FI within at least 20 spheroids per condition. As shown in the graph, there is a clear increase in the FI with longer incubation times, indicating a higher amount of NBs penetrating within the spheroids. Moreover, when compared to the absence of fuel for longer incubation times, active swarms of NBs allow for a 3.2-fold enhancement in FI. The penetration of NBs can be clearly visualized in the representative microscopy image shown in Figure 6C-i, where the Cy5 signal is present even in the central regions. Moreover, the z-stack images of Figure 6C-ii showing different focal planes of the spheroid demonstrate that, despite the fact that there are NBs still present at the edges, the penetration and distribution of NBs occur throughout the entire spheroid volume. The control without fuel is shown in Figure S5A in the Supporting Information.

Previous studies using NBs have demonstrated that motion facilitates better penetration in 3D spheroids when powered by inorganic catalysts,^35^ powered with enzymes like urease^36^ or collagenase,^37^ and even photothermally driven.^38^ These previous works reported an average increase of about 2.6-fold in NBs internalizations. Of particular interest is a previous study by Hortelao et al., which used mesoporous silica urease-powered NBs to evaluate internalization through a 4 h incubation in the same cell type (3D spheroids of RT4 cells).^36^ Despite the significant differences in incubation time (our experiments were only performed for 20 min), the enhancements reported by Hortelao et al. were in the range of 3.5-fold, which is similar to the enhancements reported here using LBL PLGA NBs which is 3.2-fold. As discussed in the previous section regarding enhancements in the delivery of 2D cell cultures, the enhancements observed for the LBL PLGA NBs reported here can be attributed to the formation of vigorous swarms that lead to a better distribution of particles due to the flows generated in the process, which mediate internalization.

Lastly, we evaluated whether the enhanced internalization resulting from the collective behavior of swarming could improve the transfection of pDNA. Figure 6D presents representative fluorescent microscopy images of transfected RT4 spheroid expressing eGFP (green signal) 24h after treatment with 0.02 mg of pDNA-loaded LBL PLGA NBs. The images clearly demonstrate the expression of eGFP, confirming the successful internalization and intracellular release of pDNA. Moreover, we evaluated the eGFP expression at different depths of the spheroid (Figure 6E). Z-stack depicts how the distribution of transfected cells changes along the spheroid, confirming successful transfection along the spheroid volume as compared to the control without the fuel (Figure S5B in the Supporting Information). This study not only represents the first evaluation of the delivery efficiency of swarms of NBs, but also the first report on the delivery of pDNA using NBs.

## Conclusions

Overall, in this study, we present the design of enzyme-powered NBs that are loaded with pDNA. These NBs enable rapid and efficient delivery and transfection of cells in 2D cell cultures. When these NBs are used as swarms, they show significant improvements in terms of delivery and transfection efficiency compared to inhibited particles or control groups without fuel. In addition, experiments demonstrate that NBs are able not only to overcome the limitations of passive nanocarriers for penetration of 3D spheroids (extracellular barrier) but also to allow successful intracellular delivery by surpassing endolysosomal degradation of the nucleic acids. Furthermore, the materials used in the design of these NBs are biocompatible and biodegradable, making them promising candidates for clinical applications, such as gene-based therapies for urinary tract malignancies.

## Experimental Section/Methods

### Materials

PLGA of 24–38 kDa and composition 50:50 (BLDpharm); Mowiol of 31 kDa (Thermo), ethyl acetate anhydrous 99.8% (Sigma); Cyanine5 NHS ester (Lumiprobe); low-molecular-weight chitosan, branched polyethylenimine of 25 kDa, glutaraldehyde grade II (Glu, 25% in H_2_O), and urease from *Canavalia ensiformis* Type IX 50,000–100,000 units g/g solid were all purchased from Sigma-Aldrich. PBS, Dulbecco’s modified Eagle’s medium (DMEM), and McCoy’s 5A (modified) medium were purchased from gibco.

### Plasmid Extraction

The plasmid gWIZ eGFP-pDNA (Promega, Leiden, The Netherlands), was amplified in transformed *Escherichia coli* bacteria and isolated from the bacteria suspension using the kit Plasmid Plus Giga Kit (Qiagen), following the protocol recommended by the manufacturer. Concentration and purity were determined on a NanoDrop 2000c instrument (Thermo Fisher Scientific).

### PLGA NP Synthesis

PLGA nanoparticles were synthesized by an oil-in-water emulsion (OW) method. The protocol was adapted from Haque et al.^39^ Briefly, 10 mL of an aqueous solution of 100 mg of Mowiol MW 31 kDa and 30 mg of low-molecular-weight chitosan 50–190 kDa was sonicated for 20 min and stirred for 10 more minutes until complete dissolution. Then, 30 mg of PLGA MW 24–38 kDa and composition 50:50 was dissolved in 9 mL of anhydrous ethyl acetate 99.8%. The oil-in-water emulsion was precisely controlled by adding the PLGA solution (organic phase) into the Mowiol solution (aqueous phase) using a 21G needle, a flow rate of 5 mg/mL, and stirring at 200 rpm. After the addition, the sample was homogenized using a 3 mm tip sonicator (Branson) for 1 min at 55% intensity. Finally, the organic solvent was evaporated by letting the sample stir (200 rpm) in an open flask overnight (21 h). Labeled PLGA nanoparticles were obtained by adding 20 μL of Cyanine5 NHS ester (stock: 5 mg/mL in 25% DMSO, 75% MQ water) after the tip sonication step.

### LBL PLGA NB Synthesis

PLGA NPs with CS were used as the first layer. To load negatively charged pDNA onto the PLGA NPs (second layer), 500 μL of 1 mg/mL PLGA NPs were collected in a 1.5 mL low protein binding tube (Thermo Scientific) by centrifugation at 3500 rcf for 10 min at 4 °C. The pellet was resuspended in HEPES (10 mM, pH = 7) and different amounts of pDNA (1–100 μg) were added (final volume 500 μL). Incubation with pDNA was kept for 2 h in a Thermomixer Comfort (eppendorf) under shaking at 1250 rpm. Maximum loading, and thus the conditions to proceed with the attachment of the third layer, was achieved by adding 80 μg of pDNA (160 μg of pDNA per milligram of PLGA). Particles were washed by centrifugation, as described for the previous layer. To obtain the final PEI layer, the pellet was resuspended in 450 μL of PBS and again 50 μL of PEI (0.1% v/v) was added and incubated for 30 min under shaking. LBL PLGA NPs were washed by centrifugation, as described for the other layers.

For the NB synthesis, the pellet of LBL PLGA NPs was resuspended in 480 μL of H_2_O MQ and 20 μL of glutaraldehyde (1:10 diluted in MQ from stock) was added to the mixture. The mixture was shaken for 2 h to activate the amine groups of the third layer of PEI. Then, the particles were collected by centrifugation (10 min, 3500 rcf). Next, the pellet was suspended in 500 μL PBS containing urease (final urease concentration = 3 mg/mL) and mixed for 24 h in an end-to-end on a rotary shaker. The resulting LBL PLGA NBs were collected by centrifugation as described for the previous steps and resuspended in 50 μL of PBS for further experiments.

For the physicochemical characterization, NBs were washed and resuspended in MQ water, after which they were transferred either into a disposable folded capillary cell (Malvern, Worcestershire, U.K.) or into a disposable cuvette (Brand, Wertheim, Germany) for further measurement of their ζ-potential or hydrodynamic size, respectively, using a Malvern Zetasizer Nano (Malvern Instruments Ltd., Worcestershire, U.K.). The measurements were performed in triplicate at a temperature of 25 °C.

For the FTIR characterization, dried samples were measured in a Nicolet iS 10 FTIR spectrometer in the spectral range 4000 to 525 cm^–1^.

Gel electrophoresis experiments were performed using a 1% agarose gel made in 1× Tris-Acetate-EDTA (TAE) buffer (40 mM Tris, 20 mM acetic acid, and 1 mM EDTA, pH 8) with GelRed Nucelic Acid Stain (Sigma-Aldrich).

Inhibited NBs were generated by incubation of LBL PLGA NBs for 1h in the presence of 1 mM acetohydroxamic acid, which inhibits the hydrolysis of urea reversibly. After incubation, particles were centrifuged and directly applied.

### Single-Particle Motion and *In Vitro* Swarming Analysis

Observation and video recording of the NBs were performed in a THUNDER optical microscope (Leica) using a 100× water objective. Briefly, 5 μL of NBs in PBS were placed on the center of a 9 mm diameter and 0.12 mm deep Secure-Seal spacer (Thermo Fisher Scientific) stuck onto a glass slide and thoroughly mixed with the solutions of urea in PBS at the desired concentrations. Then, the mixture was covered using a coverslip to avoid artifacts caused by the drifting effect. Videos of 30 s were recorded using a Hamamatsu camera at a frame rate of 50 fps under bright field. The analysis of motion was performed with a homemade Python code to obtain the tracking trajectories, MSD, and diffusion coefficients as previously described.^16^ The resulting MDS and diffusion coefficients were obtained by analyzing a minimum of 15 particles per condition, and the error represents SE.

The optical videos of the swarms of NBs were acquired using a THUNDER Leica microscope using a 2.5× objective. For this, a 0.5 μL (0.005 mg) or 2 μL (0.02 mg) droplet of NBs suspended in PBS was placed in the middle plane of a 35 mm glass-bottom μ-Dish (IBIDI) containing 3 mL of either PBS (control) or a solution of 50 mM or 100 mM urea in PBS. After addition of the drop, 2 min videos were acquired at a frame rate of 25 frames per second as previously reported.^6^

For calculating the cumulative area of the NB swarms, first, the background was subtracted from each frame of the video. As a background image, a snapshot was taken before NBs were injected into the Petri dish. Then, an intensity threshold of 10% of the global maximum intensity value was used for all of the samples. Pixels of intensity values above this threshold are considered as the area occupied by the NB swarm. Using this threshold, the pixel intensity *I* of pixel number *n* in frame number *i* of the video is converted into a binary image using
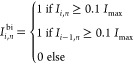
1where *I*_max_ is the global maximum intensity of a pixel in the video.

The area is then calculated as the sum of the binary pixel intensities
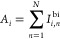
2with *N* being the total number of pixels.

Alternatively, for experiments performed in the presence of 50 mM urea in PBS, the analysis was performed without previously taking a snapshot of the background. For this, a custom-made Python script based on computer vision was developed to identify the background and subtract it from each frame. The open-source computer vision library OpenCV version 4.8.1 was used.

### Cell Culture

MB49 cells (murine urothelial carcinoma cell line) were cultured in Dulbecco’s modified Eagle’s medium (DMEM) containing d-glucose, l-glutamine, and sodium pyruvate. Full cell medium was prepared by adding 10% FBS and penicillin-streptomycin as supplements. Cells were seeded at a density of 300k cells/dish in the μ-Dish (IBIDI) specified in the swarming analysis section and incubated for 24 h at 37 °C, 5% CO_2_ prior to treatment. MB49 cell line was derived from an adult C57BL/6 male mouse. This cell line has lost the Y-chromosome and therefore does not express male-specific antigens, which is a frequent early event in bladder cancer. Cells were used up until passage 20 in each experiment.

The human bladder cancer cell line RT4 was cultured in McCoy’s 5A medium, supplemented with FBS (10%) and penicillin-streptomycin solution, at 37 °C in a humidified atmosphere of 5% CO_2_. The cells were split every 2 days in a 1:4 ratio. To obtain 3D RT4 cell cultures, cells were seeded evenly at a density of 75 × 10^3^ cells per cm^2^ and were allowed to grow for 3 days before the experiments, with the medium changed every 2 days. RT4 cells exhibit epithelial morphology and were isolated from urinary bladder tissue derived from a 63-year-old, white, male patient with transitional cell papilloma.

### Viability in MB49 Cells

Viability was assessed after treatment using the CellTiter-Glo luminescent cell viability assay, as recommended by the manufacturer (Promega, Leiden, The Netherlands). Briefly, MB49 cells were seeded at 45,000 cell/well in a 96-well plate 24h prior to experiments. To assess the toxicity associated with the concentration of PLGA NBs, cells were incubated for 4 h with increasing concentrations of nanobots (0.0001–0.6 mg) in unsuplemented cell medium (final volume = 100 μL). After incubation, cells were washed with PBS and incubated for 24h in fresh full cell culture medium. For the viability quantification, cells were supplemented with an equal volume of CellTiter-Glo reagent for each well (100 μL), mixed for 10 min using an orbital shaker (120 rpm), and transferred to an opaque 96-well plate. After allowing the plate to stabilize for 10 min, the luminescent signal of each well was measured using a SPARK multimode microplate reader (TECAN).

The effect of the enzymatic reaction, and the production of possible toxic subproducts, was also evaluated by incubation of 0.02 mg of PLGA NBs for different selected time periods (10, 20, or 30 min) in the presence of increasing concentration of urea in PBS (0, 50, 100, 200, or 300 mM). After incubation, cells were washed with PBS and incubated for 2 h in fresh cell medium prior to quantification as described above.

### Evaluation of Delivery Efficiency and eGFP Transfection with Swarms of LBL PLGA NBs in 2D Cell Cultures

MB49 cells were seeded (300k cells/dish) 24 h prior to every delivery experiment. A typical delivery experiment consisted of filling the dish with 3 mL of 50 or 100 mM urea in PBS or simply PBS, prior to seeding 0.5 μL (0.005 mg) or 2 μL (0.2 mg) of NBs at the center of the dish. After a certain incubation time (1, 10, or 20 min), cells were washed with PBS, new medium was added, and the cells were left to recover from treatment for 2 h prior further analysis. Lipofectamine 3000 Transfection kit (ThermoFisher) was used as a benchmark following the manufacturer’s instructions and working at the same effective concentration of pDNA (3.2 μg) as in the experiments performed with 0.2 mg of NBs.

For flow cytometry measurements, cells were trypsinized, collected by centrifugation (500 rcf, 3 min), and resuspended in 100 μL of PBS containing DAPI (1:1000 dilution from a 1 μg/mL stock). This last one was used as a colorimetric indication of dead cells in the flow cytometry measurements. For the delivery visualization, instead of being trypsinized, cells were first incubated with Hoechst33342 (1000×) for 10 min at 37 °C. After staining, the cells were washed with culture medium and directly imaged in glass-bottom dishes after treatment by a THUNDER optical microscopy (Leica). Images were analyzed using the ImageJ software (FIJI, https:/Fiji.sc/) to visualize and quantify the MFI associated with the presence of Cy5-labeled NBs.

Cell viability was assessed in parallel using the CellTiter-Glo reagent. For this, cells were trypsinized and resuspended in 1 mL of cell medium. From this suspension, 100 μL was placed in a 96-well plate and supplemented with an equal volume of CellTiter-Glo reagent for each well (100 μL). We proceeded to quantify the cell viability as described above.

### Flow Cytometry

Quantifications based on fluorescence were performed using a 4 laser spectral Aurora flow cytometer (Cytek). The resulting flow cytometry data were analyzed using FlowJo (Treestar, Inc., Ashland) software.

### Evaluation of Delivery Efficiency and eGFP Transfection with Swarms of LBL PLGA NBs in 3D Spheroids

RT4 cells were seeded at a density of 75 × 10^3^ cells per cm^2^, 72 h prior experiments. A typical experiment consisted of filling the dish with 3 mL of 100 mM urea in PBS or simply PBS, prior to seeding 0 2 μL (0.2 mg) of NBs at the center of the dish. After a certain incubation time (1, 10, or 20 min), cells were washed with PBS, new medium was added, and the cells were left to recover from treatment for 24 h prior further analysis. For the delivery or eGFP expression visualization, cells were directly imaged in a THUNDER optical microscopy (Leica). Images were analyzed using the ImageJ software (FIJI, https:/Fiji.sc/) to visualize and quantify the MFI associated with the presence of Cy5-labeled NBs.

### Statistical Analysis

All data are shown as the mean ± standard deviation. Statistical differences were analyzed using GraphPad Prism 8 software (La Jolla, CA). The statistical tests used in each figure are listed in the figure caption. Statistical differences with a *p*-value <0.05 were considered significant.

## Supporting Information Available

LBL FTIR characterization; motion analysis of LBL PLGA NBs for increasing concentration of urea in PBS; *in vitro* swarming of LBL PLGA nanobots in the presence of 50 mM urea in PBS; effect of swarming on the delivery enhancement between active and inhibited particles in MB49 cells; microscopy images of the delivery and transfection by LBL PLGA NBs in the absence of fuel in spheroids of RT4 cells (PDF)
Swarming behavior of active LBL PLGA NBs nanobots in the presence and absence of 100 mM urea in PBS and inhibited NBs in the presence of 100 mM urea (MP4)
Evolution of the covered area in the presence and absence of 100 mM urea in PBS and inhibited NBs in the presence of 100 mM urea (MP4)
Swarming behavior of active and inhibited LBL PLGA NBs nanobots in the presence of 50 mM urea in PBS (MP4)
Evolution of the covered area of active NBs and inhibited NBs in the presence of 50 mM urea (MP4)

## Abbreviations

NBs: nanobots
DDS: drug delivery system
NPs: nanoparticles
LBL: layer-by-layer
PLGA: poly(lactic-*co*-glycolic acid)
FDA: Food and Drug Administration
EMA: European Medicines Agency
pDNA: plasmid DNA
mRNA: mRNA
siRNA: short interfering RNA
CS: chitosan
PEI: polyethylenimine
eGFP: green fluorescent protein
DLS: dynamic light scattering
PDI: polydispersity index
MSD: mean squared displacement
NTC: nontreated cells
rMFI: relative mean fluorescence intensity
