# Supplementary material for: Swarms of Enzymatic Nanobots for Efficient Gene Delivery
Source: ACS Appl Mater Interfaces. 2024 Aug 27;16(36):47192–205. doi: 10.1021/acsami.4c08770 (PMC11403613; doi:10.1021/acsami.4c08770)
Supplement: Supplementary file 1 — am4c08770_si_001.pdf [file am4c08770_si_001.pdf]

SUPPORTING INFORMATION

Swarms of enzymatic nanobots for efficient gene delivery

Juan C. Fraire,[1] Carles Prado Morales,[1] Ana Aldaz Sagredo,[1] Ainhoa G. Caelles,[1] Florencia Lezcano,[1] Xander Peetroons,[1] Anna C. Bakenecker,[1,#] Valerio Di Carlo,[1] Samuel Sánchez,[1,2*]*

[1]Institute for Bioengineering of Catalonia (IBEC), Barcelona Institute of Science and Technology (BIST), Baldiri i Reixac 10-12, 08028 Barcelona Spain.

[2]Catalan Institute for Research and Advanced Studies (ICREA), Passeig de Lluís Companys 23, 08010, Barcelona, Sapin.

[#]Current Affiliation: Fraunhofer Research Institution for Individualized and Cell-Based Medical Engineering (IMTE), Mönkhofer Weg 239a, 23562 Lübeck, Germany.

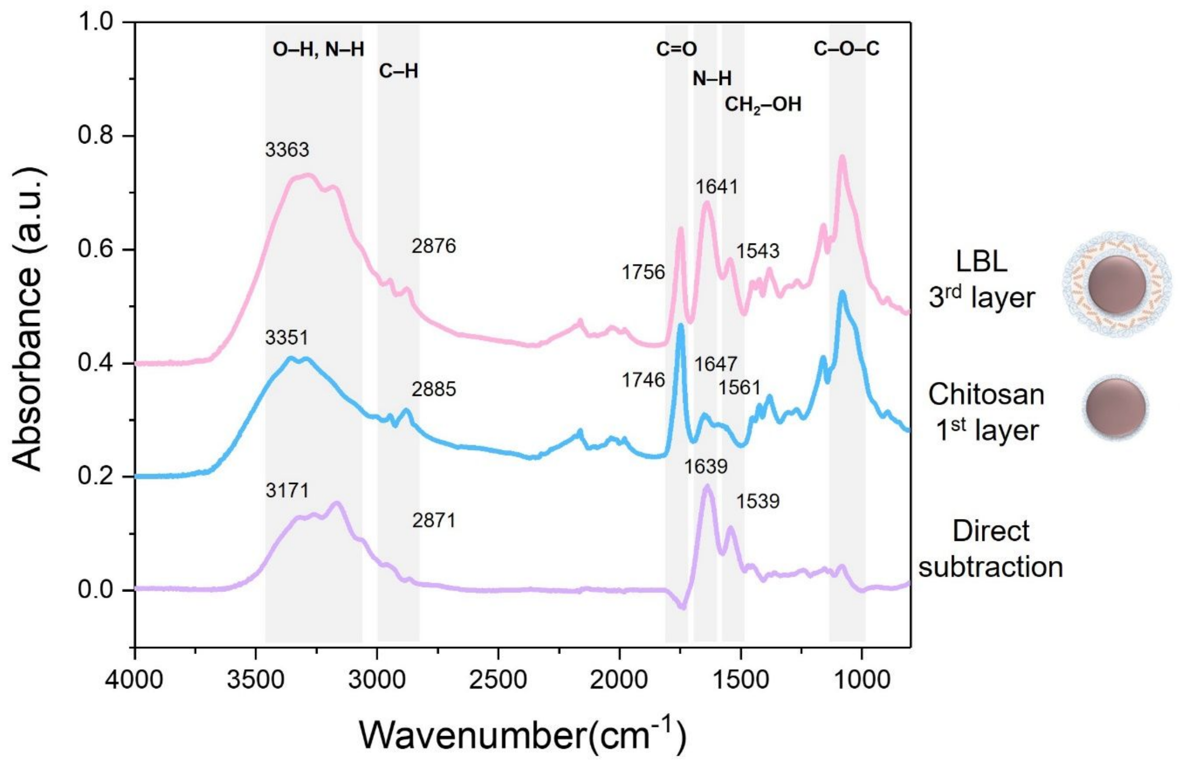

Figure S1. IR spectra of LBL PLGA NPs. From top to bottom: LBL NPs, chitosan coated PLGA NPs, direct subtraction of the spectra presented above.

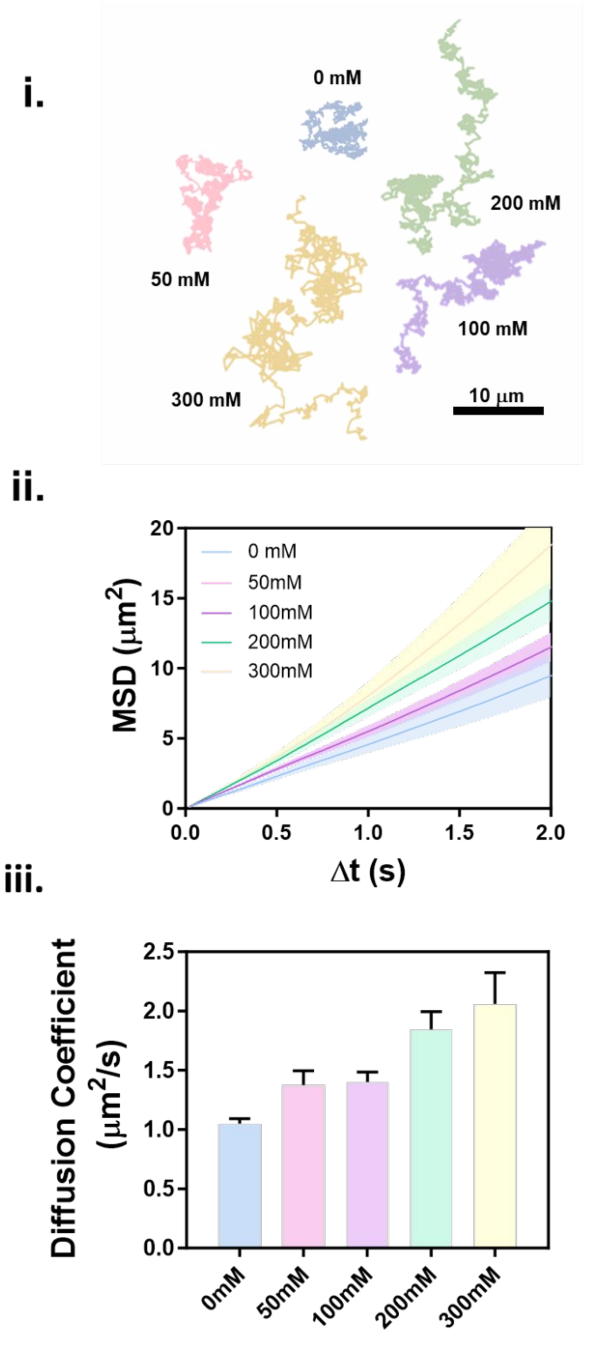

Figure S2. Single particle motion analysis of LBL PLGA motors. Motion analysis at different urea concentrations: i) representative tracking trajectories of the nanobots during 15s; ii) nanobots MSD (N = 15, error bars represent s.e.m.); iii) diffusion coefficient of nanobots obtained by optical tracking (N = 15; error bars represent s.e.m.).

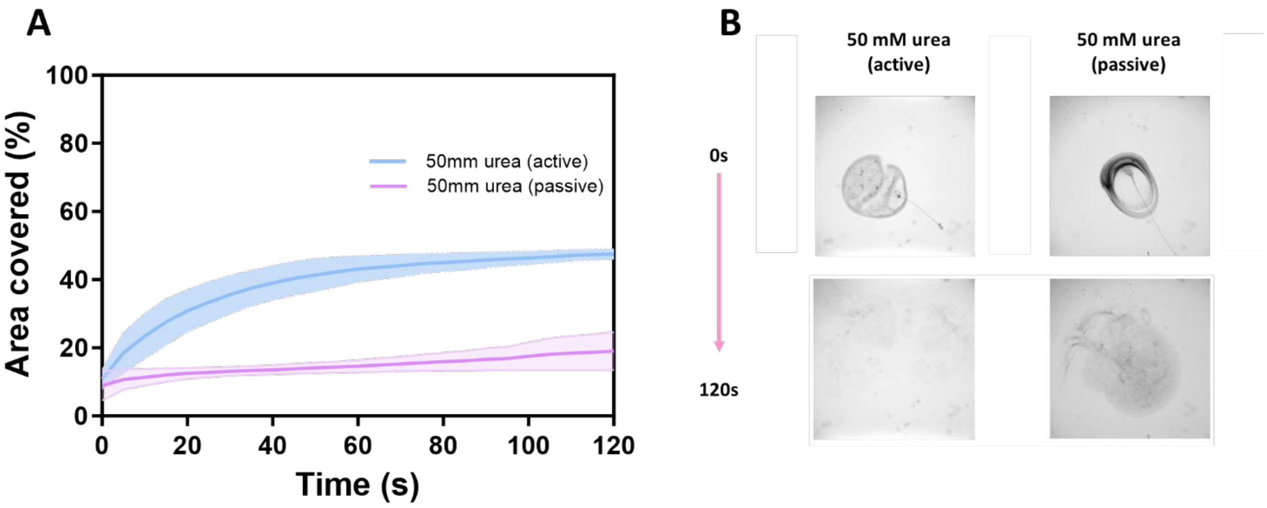

Figure S3. *in vitro* swarming of LBL PLGA motors in the presence of 50 mM urea in PBS. A) Effective area covered by the swarm cloud as a function of time performed and B) Snapshots of the *in vitro* swarming at the indicated time points (0 and 120s).

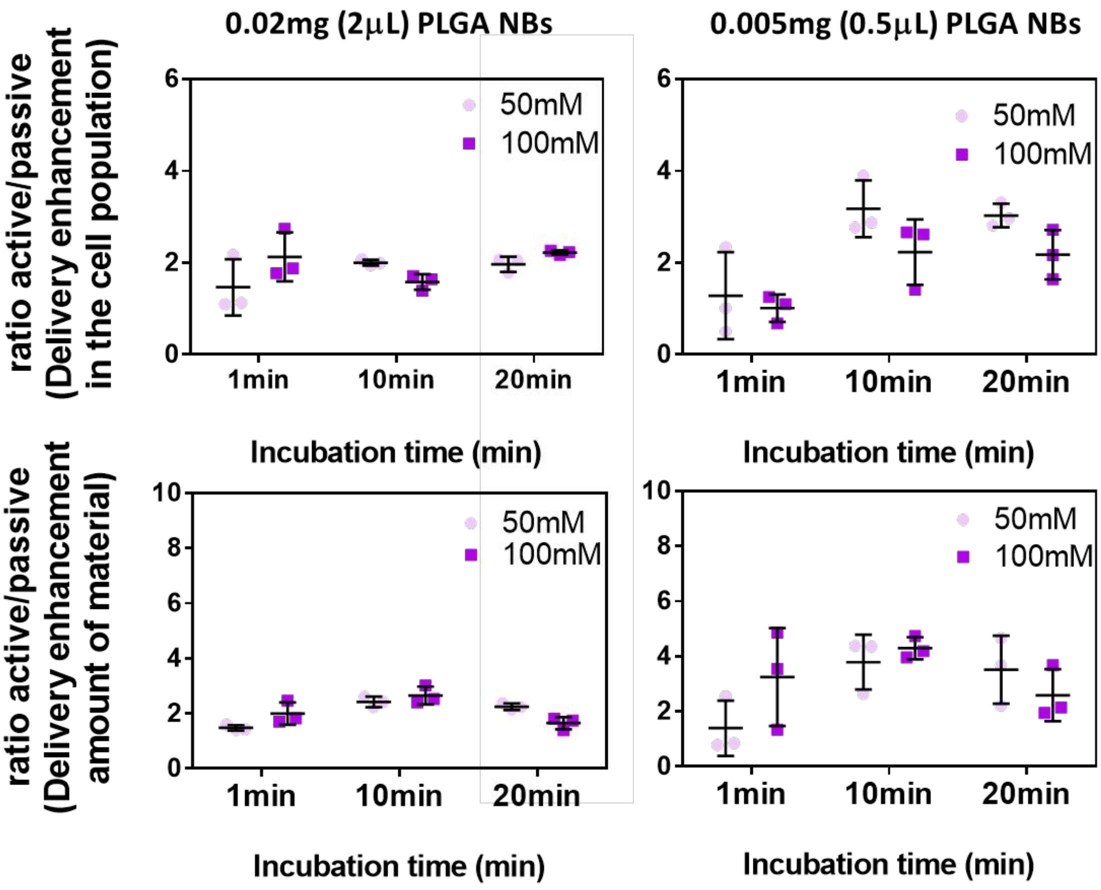

Figure S4. Comparison of the effect of swarming on the delivery enhancement between active and inhibited particles in MB49 cells (2D culture). The delivery efficiency for each condition was calculated as the ratio active/inhibited regarding: (i) the % of positive cells for the presence of Cy5 labeled NBs in the whole cell population; (ii) the amount of delivered material (rMFI).

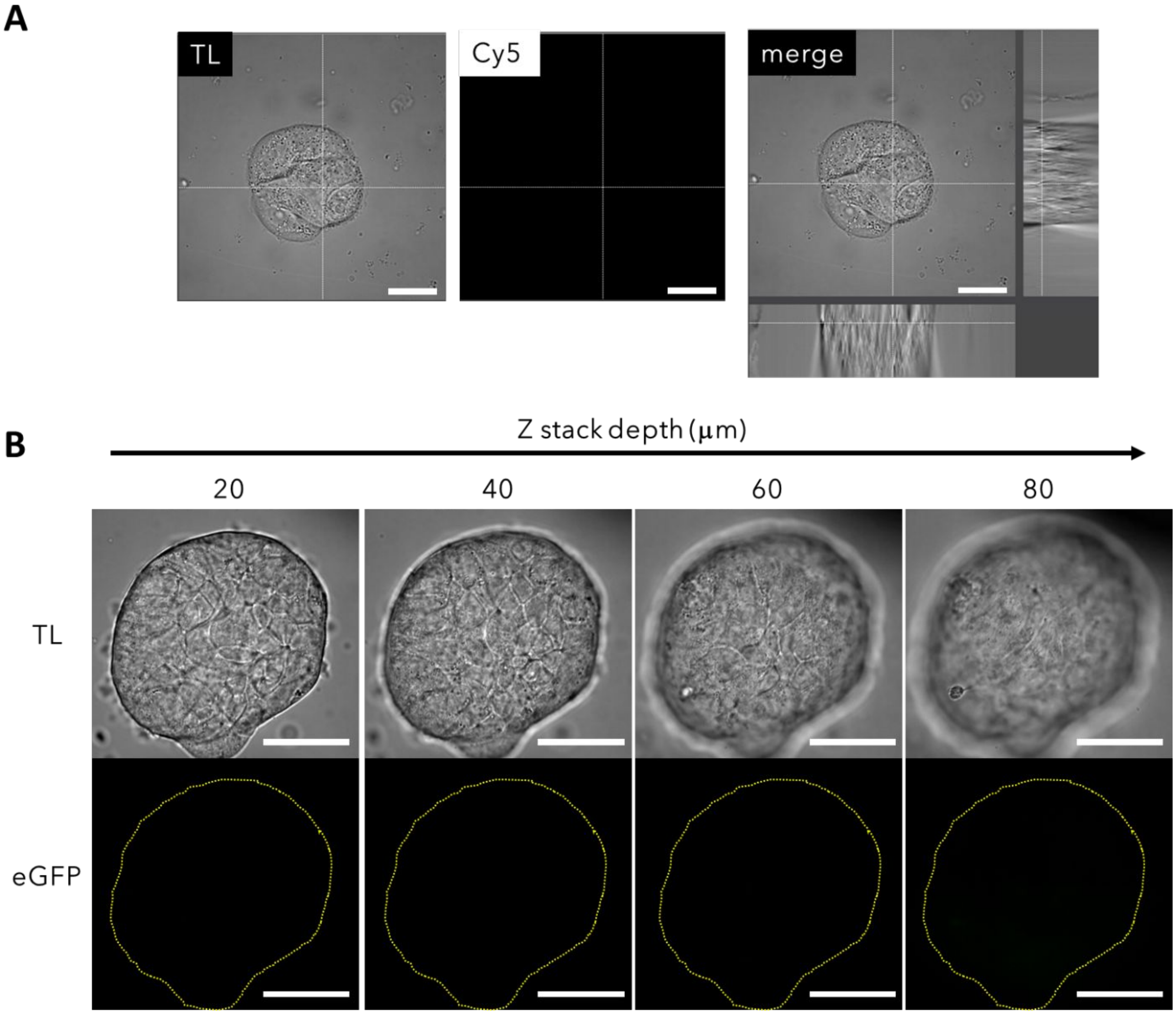

Figure S5. Delivery and transfection by LBL PLGA NBs in the absence of fuel in spheroids of human urinary bladder derived RT4 cells (3D). A) Microscopy images of RT4 spheroids after 20 min treatment with 0.02 mg of Cy5 labeled NBs in PBS without urea. The scale bar corresponds to 100 μm. B) Fluorescent microscopy images of the z-stack of a RT4 spheroid 24h after treatment with 0.02 mg of pDNA loaded LBL PLGA NBs in PBS without urea. The scale bar corresponds to 100 μm.
